# Clinical characteristics linked to persistent and emerging eating disorder risk trajectories in young people seeking mental health care

**DOI:** 10.1007/s00787-026-02978-9

**Published:** 2026-03-17

**Authors:** Ashlee Turner, Ian B Hickie, Sarah Maguire, Emiliana Tonini, Haley M LaMonica, Mathew Varidel, Alissa Nichles, Natalia Zmicerevska, Jan Scott, Christos Pantelis, Barnaby Nelson, Patrick D McGorry, Stephen J Wood, Alison R Yung, Rosemary Purcell, Elizabeth M Scott, Frank Iorfino

**Affiliations:** 1 Brain and Mind Centre, The University of Sydney, Sydney, NSW Australia; 2 InsideOut Institute for Eating Disorders, Central Clinical School, Facultyof Medicine & Health, The University of Sydney, Sydney, NSW Australia; 3 Academic Psychiatry, Newcastle University, Newcastle upon Tyne, UK; 4 Department of Psychiatry, Sunshine/Western Health, The University of Melbourne, St Albans, VIC Australia; 5 Monash Institute of Pharmaceutical Sciences (MIPS), Monash University, Parkville, VIC Australia; 6 Florey Institute of Neurosciences of Mental Health, Parkville, VIC Australia; 7 Orygen, Parkville, VIC Australia; 8 Centre for Youth Mental Health, The University of Melbourne, Parkville, VIC Australia; 9 School of Psychology, University of Birmingham, Edgbaston, UK; 10 Institute of Mental and Physical Health and Clinical Translation (IMPACT), Deakin University, Geelong, VIC Australia

**Keywords:** Early intervention, Risk trajectories, Youth mental health, Disordered eating behaviours, Psychopathology, Help-seeking youth

## Abstract

Eating disorders (EDs) are serious mental health conditions with peak onset during adolescence and young adulthood. While early intervention services frequently encounter young people at-risk of EDs, little is known about the trajectories and factors associated with ED risk among those engaged in services over time. The sample includes 494 young people (aged 12–25 years) who presented to youth mental health services in Australia between 2011 and 12, primarily for emerging mood disorders. ED risk was assessed at baseline and 12-month follow-up using the SCOFF questionnaire, with scores *≥* 2 (range 0–5) indicating high risk status. Participants were classified into four trajectory groups: persistent (high risk at both timepoints; *n* = 92, 18.62%), remitting (high to low risk; *n* = 69, 13.97%), emerging (low to high risk; *n* = 51, 10.32%), or low risk (low risk at both timepoints; *n* = 282, 57.09%). Clinical factors included depression, anxiety, deliberate self-harm, functioning, and rumination. Logistic regression analyses were used to identify factors associated with persistent risk (vs. remitting risk) and emerging risk (vs. low risk). ED risk status showed significant individual stability over time (75.71% maintained their baseline status, *p*<.001). Greater rumination was associated with worse outcomes in both regression analyses (i.e., persistence vs. remitting risk, and emergence vs. low risk; all *p*<.05). These findings suggest that rumination may represent a key modifiable risk factor that could inform targeted interventions in youth mental health services. The substantial proportion of young people with persistent or emerging ED risk highlights a significant unmet need in youth mental health services and intervention strategies are needed to improve long-term outcomes for this population.

**Supplementary Information:**

The online version contains supplementary material available at 10.1007/s00787-026-02978-9.

## Introduction

Youth mental health has been labelled a global crisis [1]. One in ten young people aged 5–24 are currently living with a mental disorder [2], with 75% of adult mental illness first emerging before age 25 [3]. These challenges represent a leading cause of disease burden in youth [4], with profound implications for social relationships, school and work attainment, and physical health [5, 6]. Mental health challenges during youth, even when subthreshold or temporary, can significantly disrupt the transition to adulthood and lead to persistent impairments [7]. The rising prevalence and substantial impacts of these challenges underscore the critical need for early identification and youth-focussed intervention systems.

Within the broader youth mental health landscape, eating disorders (EDs) and disordered eating behaviours represent a particularly concerning and complex challenge that demands urgent attention [8]. Globally, the prevalence of EDs has risen steadily over recent decades [9]. In Australia, it is estimated that approximately 1.1 million people are impacted by EDs, with young people being disproportionately affected compared to other ages groups [10]. Studies have estimated that 16–22% of young people have a diagnosed ED [11, 12], and over one-quarter report subclinical disordered eating behaviours [13]. The development and maintenance of EDs involves a complex interplay of biological (e.g., genetic vulnerability), psychological (e.g., comorbid mental health conditions, body image concerns and body dissatisfaction, personality traits) and environmental and sociocultural risk factors (e.g., family and peer relationships, childhood adversity, exposure to ‘thin appearance ideals’ and diet culture) [14]. While common risk factors are shared, females are more likely to experience an ED compared to males and presentations tend to differ between sexes [10, 11]. The peak onset of EDs spans adolescence into young adulthood, a critical period when these disorders can significantly disrupt cognitive, educational, and socio-emotional development [15]. EDs are also linked to a range of physical and mental health comorbidities including depression and anxiety, greater risk of self-harm and suicide attempts, lower functioning, increased mortality, and poorer quality of life [16, 17]. Despite these serious consequences, lengthy untreated illness durations are common amongst individuals with EDs [18], leading to poorer treatment outcomes over time [19]. Given the wide-ranging adverse effects of EDs in this critical life stage, early intervention is imperative to prevent long-term disability and has been highlighted as one (of eight) ED treatment principles in Australia [20].

In Australia, the *headspace* early intervention service and model of care aims to address the early stages of mental disorders in young people with varying needs. Similar early intervention youth mental health service models exist internationally [21–23]. As an enhanced primary care-based service, it focusses on providing mental health assessment and appropriate psychological, psychosocial and medical interventions for any young person, with the goal of preventing or delaying illness progression [24, 25]. While most young people present to *headspace* for depressive symptoms and anxiety [26], these disorders frequently co-occur with EDs [27]. Recent data show that nearly half of those seeking care at one *headspace* centre in metropolitan Sydney report significant body image concerns, and more than one-third engage in disordered eating behaviours [28]. Although many people do not actively seek help due to the ego-syntonic nature of EDs, denial or lack of awareness of illness severity, or the stigma associated with them [29, 30], these disorders may also go undetected or unaddressed in services when anxiety and mood concerns are the primary focus of care. Despite the increased risk of EDs among young people presenting to early intervention mental health care services, little is known about the course and patterns of risk among those engaged in these services over time.

Understanding the changes in ED risk over time, along with the predictors of different illness trajectories, in real-world clinical cohorts is critical for developing mental health services that cater to the individual needs of young people. This study aimed to identify ED risk trajectories among of young people attending primary mental healthcare services and examine identify the demographic and clinical characteristics associated with differing trajectories using data from the *Transitions* cohort [31], a longitudinal prospective multi-site clinical study.

## Methods

The protocol for the *Transitions* study was approved by the Human Research Ethics Committees at the University of Melbourne and the University of Sydney. The detailed protocol for this study and the baseline characteristics of the sample have been published elsewhere [31, 32].

### Participants and procedure

From January 2011 to August 2012, 802 young people aged 12–25 years who presented to one of four *headspace* clinical services in Sydney and Melbourne consented to participate in a prospective longitudinal cohort study investigating the course of mental disorders among young people. Participants were excluded if they had a significant intellectual disability (e.g., clinically assessed IQ < 65), were non-English speaking and/or were unable to complete assessments in English or were unwilling or unable to provide informed consent. Young people who were acutely suicidal were not approached for inclusion until their treating clinician confirmed that their suicidality had resolved to the point of no longer being at high risk. Written informed consent was obtained from participants aged *≥* 15 years, whereas those aged 12–14 years (inclusive) provided assent with parent/guardian written informed consent.

Data collection was conducted in person at the *headspace* clinics by trained research assistants who held graduate qualifications in psychology. Following structured clinical interviews, participants completed self-report questionnaires using electronic devices (iPads or laptops) provided by the research team. Follow-up assessments using the same measures were conducted at the *headspace* clinics at 12 months post-baseline, with data collection concluding in December 2013. Participants were compensated with a $20 gift voucher for their time.

### Measures

*Eating disorder risk.* The SCOFF is a 5-item self-report tool that screens for symptoms of EDs to identify individuals who may be at-risk for anorexia nervosa and bulimia nervosa based on endorsement of key symptoms of illness [33]. The acronym is derived from the five items, which ask about (1) making yourself **s**ick, (2) worry about losing **c**ontrol, (3) losing more than **o**ne stone (about 6.35 kg) in three months, (4) believing yourself to be **f**at, and (5) **f**ood dominating your life. Each “yes” answer scores one point and the summed total score is used to indicate a positive (total score *≥* 2, classified as ‘high risk’) or negative screen (total score < 2, classified as ‘low risk’). Previous studies have found this cut-off to be highly sensitive (72–100%) and specific (73–94%) for identifying cases of anorexia nervosa and bulimia nervosa [33, 34]. Based on SCOFF scores at baseline and follow-up, participants were classified as having: (1) emerging risk (low risk at baseline to high risk at follow-up, i.e., worsening over time), (2) remitting risk (high risk at baseline to low risk at follow-up, i.e., improving over time), (3) persistent risk (high risk at baseline and follow-up), or (4) low risk (low risk at baseline and follow-up).

*Depressive symptoms.* The Quick Inventory of Depressive Symptomatology (QIDS) is a 16-item clinician-rated inventory that measures the major diagnostic depressive symptoms [35]. Each symptom is rated on a 4-point Likert scale, with total scores ranging from 0 to 27 (higher scores indicating more severe depressive symptoms).

*Anxiety symptoms.* The Generalized Anxiety Disorder scale (GAD-7) is a 7-item self-report tool that measures core symptoms of generalised anxiety disorder [36]. Items are scored on a 4-point Likert scale, reflecting how often the individual experienced the anxiety symptom, with total scores ranging from 0 to 21 (higher scores indicating worse anxiety).

*Functioning.* The Social and Occupational Functioning Scale (SOFAS) is a single-item clinician-rated global assessment of social and occupational functioning [37]. Scores range from 0 to 100, with higher scores indicating better functioning and scores < 70 considered to be clinically-significant impairment [38].

*Rumination.* A brief 10-item self-report questionnaire was used to assess ruminative style [39], adapted from a validated measure [40]. Each item is scored on a 4-point Likert scale, with total scores ranging from 10 to 40 (higher scores indicate a higher degree of rumination about negative feelings and experiences).

*Deliberate self-harm.* Two questions were used to assess self-injurious behaviours with and without suicidal intent that have non-fatal outcomes. Participants were asked (yes/no) whether they had deliberately hurt themselves, or done something to try and kill themselves, in the past 12 months. A response of “yes” to either question was classified as deliberate self-harm [41].

### Statistical analysis

Analyses were conducted in R (version 4.4.1) [42]. Descriptive data were reported as mean ± standard deviation for continuous data and frequencies and percentages for categorical data. Baseline demographic and clinical characteristics were compared between trajectory groups using independent samples t-tests and chi-square tests. Individual stability of risk status over time was assessed using McNemar’s test and exact binomial testing against chance.

Two separate logistic regressions were conducted to explore the association of baseline clinical and demographic variables with ED risk trajectories among participants with the same baseline risk status: one comparing persistent versus remitting risk (among those classified as high risk at baseline), and another comparing emerging versus maintained low risk (among those classified as low risk at baseline). This modelling strategy aligned with distinct intervention approaches needed for different risk trajectory groups (i.e., secondary prevention for those already at high risk, versus primary prevention for those initially at low risk) [43]. The clinical variables (depressive symptoms, anxiety, deliberate self-harm, functioning, and rumination) were selected based on established theoretical and empirical associations with eating disorder risk [14, 44–46]. This approach acknowledged that different baseline factors may be relevant for high risk persistence versus emergence, and enhanced clinical interpretability by differentiating between factors that are associated with persistent high risk versus those associated with the emergence of risk over time [47]. All variables were entered simultaneously using the enter method in both regression models, with age and sex included as covariates to account for potential confounding.

## Results

**Fig. 1:**
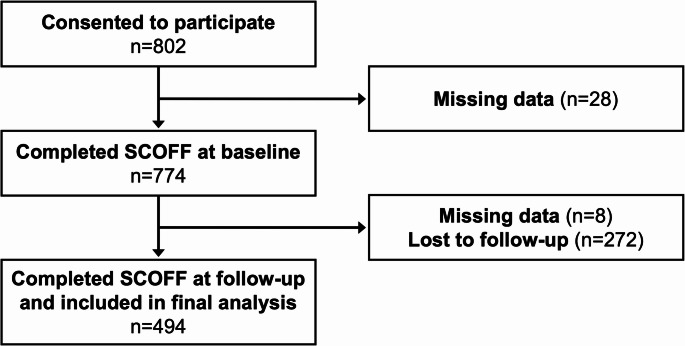
Flow diagram showing participant progression from initial consent to final analysis sample

Of the 802 participants who consented to participate in the *Transitions* study, 774 (96.51%) completed the SCOFF at baseline. Of these, 494 (63.82%) completed the SCOFF at follow-up and were included in the final analysis (Fig. 1). Sensitivity analyses comparing participants who completed follow-up assessment versus those lost to follow-up showed that males and those with lower baseline functioning were more likely to drop out, but there was no difference in attrition rates between participants with high versus low ED risk at baseline (see Supplementary Table S1).

### Demographic and clinical characteristics

Participants had a mean age of 18.35 years, were predominantly female (68.83%), and most were engaged in employment or education (83.00%) (Table 1).

**Table 1:** Demographic and clinical characteristics of participants

Characteristic	Total (*n* = 494)
*Demographic variables*	
Age, years	18.35 ± 3.21
Sex, % female	340 (68.83)
NEET, % yes	84 (17.00)
*headspace* location, % Sydney	259 (52.43)
*Clinical variables*	
Eating disorder risk	1.14 ± 1.25
Depressive symptoms	10.14 ± 5.47
Anxiety symptoms	9.73 ± 6.00
Functioning	66.37 ± 11.66
Rumination	28.95 ± 6.83
Deliberate self-harm, % yes	212 (42.91)

Note. NEET = Not in Employment, Education or Training. Eating disorder risk measured using SCOFF (0–5); Depressive symptoms measured using Quick Inventory of Depressive Symptomatology scale (QIDS, 0–27); Anxiety symptoms measured using Generalised Anxiety Disorder scale (GAD-7, 0–21); Rumination measured using brief rumination questionnaire (10–40); Functioning measured using Social and Occupational Functioning Assessment Scale (SOFAS, 0-100). Higher scores indicate greater severity except for SOFAS where higher scores indicate better functioning.

Data are presented as mean ± standard deviation for continuous variables and n (%) for categorical variables.

^φ^ Missing data for *n* = 1.

### Trajectories of ED risk from baseline to follow-up

**Fig. 2:**
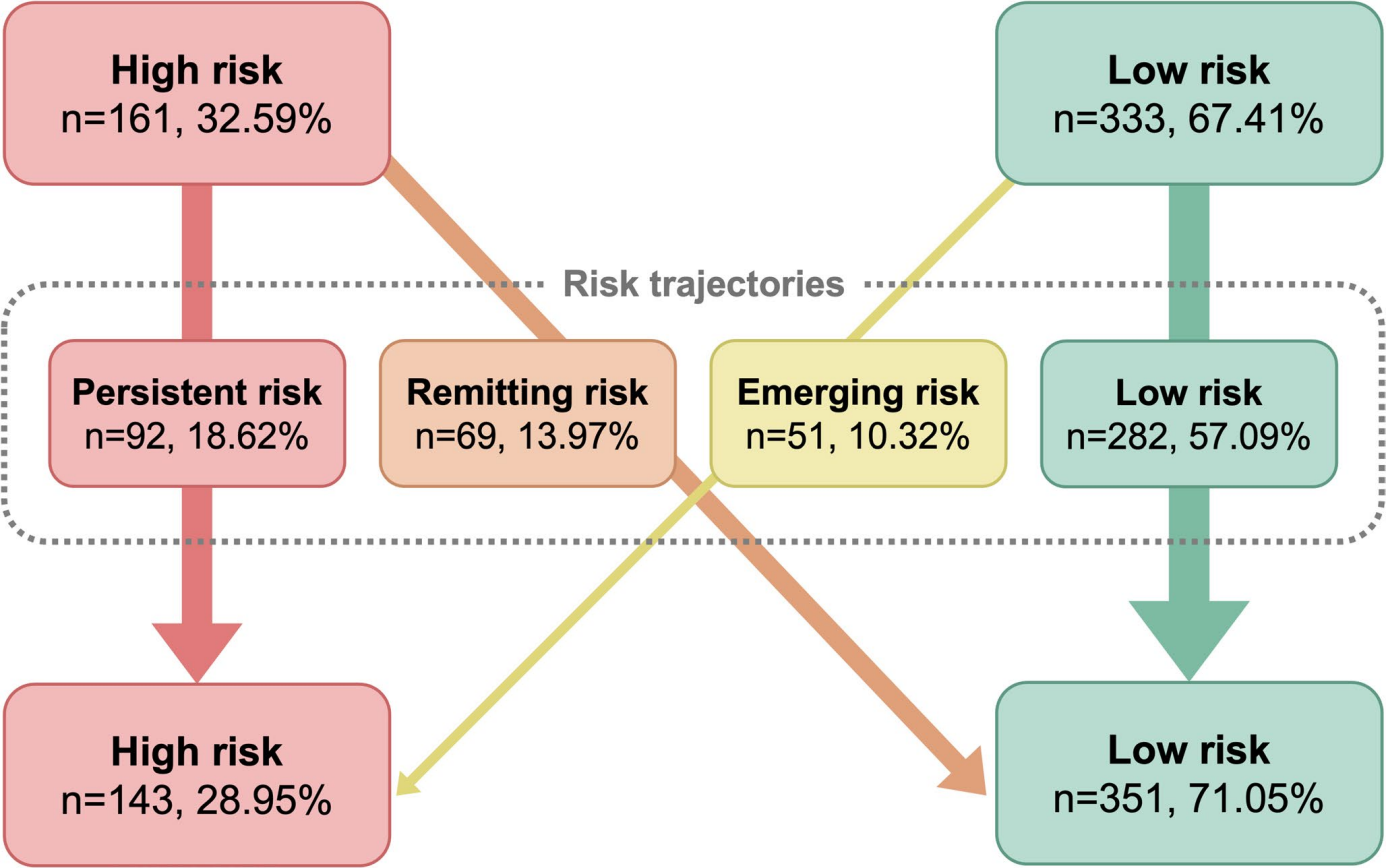
Trajectories of ED risk over 12 months. Participants were classified at baseline and 12-month follow-up as high risk (SCOFF *≥* 2) or low risk (SCOFF < 2). Four trajectory groups were identified: persistent risk (high → high), remitting risk (high → low), emerging risk (low → high), and low risk (low → low). Arrow thickness reflects group size. Numbers represent count and percentage of the total sample (*n* = 494)

Figure 2 shows the trajectories of ED risk over the 12-month follow-up period. At baseline, 32.59% (*n* = 161) of participants were classified as high risk, while 67.41% (*n* = 333) were low risk. Among those classified as high risk at baseline, 57.14% (*n* = 92) showed persistent risk, while 42.86% (*n* = 69) had remitting risk (Fisher’s exact test: *p*=.014). Among those low risk at baseline, most maintained their low risk status (*n* = 282, 84.68%) and a small proportion transitioned to the high risk category (*n* = 51, 15.32%) (Fisher’s exact test: *p*<.001). Risk status showed significant individual stability over time, with 75.71% (*n* = 374) of participants maintaining their baseline classification (*p*<.001). Transitions between risk categories were balanced, with no systematic directional bias in changes (χ^2^ = 2.41, df = 1, *p*=.121). Baseline characteristics for each trajectory group are shown in Table 2.

**Table 2:** Baseline characteristics by ED risk trajectory comparison group

Persistent vs. remitting risk			
	Persistent (*n* = 92)	Remitting (*n* = 69)	*p*-value
*Demographic variables*			
Age, years	18.48 ± 3.15	18.04 ± 3.23	0.394
Sex, % female	80 (86.96)	51 (73.91)	0.058
NEET status, % yes	15 (16.30)	16 (23.19)	0.371
*Clinical variables*			
Depressive symptoms	13.18 ± 5.34	11.00 ± 5.57	**0.013**
Anxiety symptoms	12.25 ± 6.02	11.68 ± 5.60	0.538
Functioning	62.72 ± 12.16	64.45 ± 11.00	0.346
Rumination	33.04 ± 5.44	29.94 ± 6.20	**0.001**
Deliberate self-harm, % yes	60 (65.22)	34 (49.28)	0.062
Emerging vs. low risk			
	Emerging (*n* = 51)	Low risk (*n* = 282)	*p-value*
*Demographic variables*			
Age, years	17.55 ± 3.00	18.52 ± 3.26	**0.038**
Sex, % female	38 (74.51)	171 (60.64)	0.084
NEET status, % yes	8 (15.69)	45 (15.96)	1.00
*Clinical variables*			
Depressive symptoms	10.08 ± 5.07	8.95 ± 5.15	0.149
Anxiety symptoms	9.78 ± 6.37	8.41 ± 5.65	0.156
Functioning	66.25 ± 11.48	68.05 ± 11.38	0.306
Rumination^φ^	30.14 ± 6.25	27.11 ± 6.84	**0.002**
Deliberate self-harm, % yes	20 (39.22)	98 (34.75)	0.650

Note. *NEET* = Not in Education, Employment or Training. Depressive symptoms measured using Quick Inventory of Depressive Symptomatology scale (QIDS, 0–27); Anxiety symptoms measured using Generalised Anxiety Disorder scale (GAD-7, 0–21); Rumination measured using brief rumination questionnaire (10–40); Functioning measured using Social and Occupational Functioning Assessment Scale (SOFAS, 0-100). Higher scores indicate greater severity except for SOFAS where higher scores indicate better functioning.

^φ^ Missing data for *n* = 1.

Data are presented as mean +- standard eviation for continuous variables and n (%) for categorical variables (Same as Table 1)

Significant comparisons (*p*<.05) are shown in bold.

Changes in clinical variables (depressive symptoms, anxiety symptoms, and functioning) from baseline to follow-up by trajectory group are presented in Supplementary Table S2, showing that the patterns of change generally aligned with ED risk trajectories. 

### Clinical characteristics associated with risk trajectories

Among those with high ED risk at baseline, higher rumination (OR = 1.10, 95% CI [1.02, 1.19], *p*=.011) and lower anxiety (OR = 0.92, 95% CI [0.85, 1.00], *p*=.047) were significantly associated with persistent ED risk over time (Table 3).

Among those with low ED risk at baseline, higher rumination (OR = 1.08, 95% CI [1.02, 1.16], *p*=.015) and younger age (OR = 0.90, 95% CI [0.81, 0.99], *p=.*036) were significantly associated with the emergence of high ED risk over time.

Exploratory sex-stratified regression analyses are presented in Supplementary Tables S3A and S3B.

**Table 3:** Demographic and clinical characteristics associated with risk trajectories

Factor	Persistent vs. remitting risk^#^ (*n* = 161)		Emerging vs. low risk^^^ (*n* = 333)	
	OR [95% CI]	*p* value	OR [95% CI]	*p* value
Age	1.06 [0.95, 1.19]	0.270	0.90 [0.81, 0.99]	**0.036**
Sex (Ref: Male)	2.21 [0.87, 5.87]	0.098	1.64 [0.82, 3.45]	0.170
Depressive symptoms	1.06 [0.97, 1.16]	0.201	0.98 [0.01, 9.00]	0.707
Anxiety symptoms	0.92 [0.85, 1.00]	**0.047**	0.98 [0.91, 1.05]	0.623
Functioning	0.99 [0.96, 1.03]	0.746	0.99 [0.95, 1.02]	0.372
Rumination	1.10 [1.02, 1.19]	**0.011**	1.08 [1.02, 1.16]	**0.015**
Deliberate self-harm (Ref: No)	1.43 [0.70, 2.90]	0.326	0.93 [0.48, 1.77]	0.835

Note. ED = eating disorder; OR = Odds Ratio; CI = Confidence Interval. Depressive symptoms measured using Quick Inventory of Depressive Symptomatology scale (QIDS); Anxiety symptoms measured using Generalised Anxiety Disorder scale (GAD-7); Rumination measured using brief rumination questionnaire; Functioning measured using Social and Occupational Functioning Assessment Scale (SOFAS). Higher scores indicate greater severity except for SOFAS where higher scores indicate better functioning.

^#^Persistent vs. remitting risk compares those with persistent risk to those whose high risk remitted. Reference group: Remitting risk.

^^^Emerging vs. no risk compares those who developed high ED risk to those who maintained low risk. Reference group: Low risk.

Significant factors (*p*<.05) are shown in bold.

## Discussion

The aim of this study was to identify the clinical characteristics associated with different trajectories of ED risk in young people accessing mental health care over a 12-month period. We identified four different trajectories of ED risk over time: persistent risk (18.62%), remitting risk (13.79%), emerging risk (10.32%), and low risk at both timepoints (57.09%). ED risk status showed significant stability over time, with individuals more likely to maintain their baseline status than to change (i.e., persistence of high risk was more common than remission, and maintaining low risk was more common than emergence of high risk), and balanced directional transitions between risk categories.

The persistence of high ED risk among a proportion of this sample highlights a critical gap in current treatment approaches in primary mental health care. This is perhaps unsurprising given that it is estimated that only 19–36% of individuals with an ED receive treatment [48], despite elevated health service use [49]. Of those who do receive treatment, less than half will receive interventions specifically targeting their ED [50]. This is further compounded by clinicians who lack appropriate mental health literacy for EDs [51] and inefficient pathways to specialised care, which are often overburdened and under-resourced, resulting in long waiting lists and missed treatment opportunities for young people [52]. Where ED-specific treatment is received, it predominately occurs in the primary care setting [50].

Previous research indicates that as many as one-third of young people in primary care settings report symptoms of disordered eating [28], consistent with our finding that a considerable proportion of young people accessing mental health services show concerning trajectories of ED risk over time. Primary care settings offer an opportune environment for early identification and intervention [53]; however, ED symptoms often go undetected in these settings, undermining the potential for timely and effective treatment [54]. Even in transdiagnostic youth mental health services, routine screening of ED symptoms is rare [55]. Given the substantial investment of public funds into health services such as *headspace*, and the importance of early intervention in EDs, significant service reforms are needed to adequately address the burden and unmet treatment needs affecting young people with disordered eating. This likely requires a multi-faceted approach that includes: (1) integration of standardised screening for ED symptoms and severity into routine intake practice [54]; (2) implementation of digital technologies that facilitate ongoing monitoring and measurement-based care, supporting personalised clinical decision-making and tracking of outcomes over time [56–58]; (3) upskilling clinicians to improve their knowledge, skills, and confidence in treating EDs [59]; and (4) implementation of innovative early intervention service models that are appropriate for diverse primary mental health settings and have the potential to reduce the duration of untreated illness, lead to clinically significant improvements in ED symptomatology, and reduce the need for additional intensive treatment [60–62].

Although the peak onset of disordered eating behaviours and EDs occurs during adolescence and young adulthood [15], the current findings suggest some positive and encouraging outcomes. First, most participants were classified as having low ED risk at both timepoints, which is consistent with previous findings in adolescent populations [63–66]. Second, although the change in the proportion of high risk participants baseline to follow-up was not statistically significant, a greater percentage of the sample improved (i.e., were no longer classified as high risk at follow-up) compared to those who shifted from low to high risk over time, suggesting that disordered eating and ED risk can resolve over time [63, 64]. Although disordered eating behaviours tend to worsen over time in non-clinical samples [67], this study included a sample of young people accessing mental health care, where they may have received treatment for their ED risk (either directly or indirectly). For individuals with diagnosed EDs, significant improvements in symptoms following appropriate treatment have been documented [68, 69]. However, it is important to note that rates of improvement differ depending on the specific type of symptoms and ED presentations, which may explain why some people in this study improved while others maintained an elevated ED risk over time. Moreover, symptom severity also contributes to differential rates of improvement [17]. Given that the SCOFF does not measure symptom severity, we are limited in our ability to conclude whether those who improved initially experienced less severe symptoms. Despite improvement, relapse rates in EDs are significant, particularly within the first two years post-recovery [70, 71]. Digital platforms that support continuous monitoring and care coordination could help services identify early warning signs of relapse and provide more timely intervention [72], while also enabling better integration of clinical and digital care pathways to support sustained recovery [73, 74].

In this cohort of help-seeking young people, a higher level of rumination was associated with poorer outcomes related to ED risk, including both persistence and emergence of high ED risk over the 12-month follow-up period. Rumination—a maladaptive emotional regulation strategy characterised by repetitive negative thinking—has been implicated in the development and continuation of psychopathology [75]. In the context of EDs specifically, research consistently shows that individuals with EDs exhibit higher levels of rumination compared to healthy controls and also rely more heavily on other maladaptive emotion regulation strategies rather than adaptive ones (e.g., cognitive reappraisal, problem-solving) [46, 76]. Elevated rumination has also been shown to predict disordered eating behaviours in both clinical [77] and non-clinical populations [78]. Previous research has established links between general rumination, negative affect, and subsequent disordered behaviours, suggesting that rumination may be an important cognitive mechanism through which individuals engage in dysregulated eating behaviours [79]. Specifically, negative emotions may trigger ruminative thinking patterns, which maintain and amplify emotional distress, potentially driving disordered eating as a maladaptive coping response. Importantly, our use of a general measure of rumination, rather than an eating-specific measure, suggests that this propensity for rumination represents a broader cognitive vulnerability mechanism for poorer outcomes. This aligns with transdiagnostic models of psychopathology, which highlight rumination’s transdiagnostic role, suggesting common underlying mechanisms in the development and maintenance of various mental health conditions [80]. Beyond rumination, younger age was also associated with emerging risk, aligning with established patterns of ED onset typically occurring during mid-to-late adolescence [15] and suggests particular vulnerability among younger individuals accessing mental health services. While not statistically significant, the direction of effects suggest that female sex may be associated with higher odds of both persistence and emergence of ED risk, which aligns with research showing that females are more likely to have EDs [10] and experience persisting and increasing disordered eating symptoms over time [81]. However, larger studies are needed to definitively examine sex differences in trajectory predictors.

Our findings suggest that targeting ruminative thinking patterns could be valuable in both treatment and prevention contexts. It also underscores the importance of screening for rumination in youth mental health settings to identify critical groups at-risk and targets for intervention. Rumination-focussed cognitive behavioural therapy and mindfulness-based interventions have shown promise in depression and anxiety [82], potentially through enhancing metacognitive awareness and reducing emotional reactivity. Notably, a study on a cognitive-behavioural depression prevention program, which had a strong focus on addressing repetitive negative thinking, found significant reductions in both depressive and symptoms of bulimia, with changes in depressive symptoms mediating the intervention’s effect on eating behaviours [83]. This suggests that interventions targeting rumination within existing treatment and service model frameworks for depression and anxiety may opportunistically reduce ED risk among young people who might not otherwise received ED-specific interventions. While evidence-based ED prevention programs such as The Body Project have demonstrated efficacy in reducing ED symptom severity and the risk of ED onset [84], implementing these programs into resource-constrained youth mental health service settings presents many challenges. Further research is needed to determine whether rumination-focussed interventions can effectively reduce ED risk across the full spectrum of disordered eating behaviours and potential presentations, and to identify strategies for integrating evidence-based ED prevention approaches within existing service frameworks.

The association between lower baseline anxiety and persistent ED risk was unexpected, particularly given the well-established comorbidity between anxiety and EDs [85]. This might reflect ED behaviours serving as an effective, albeit maladaptive, anxiety management strategy in this sample, resulting in lower reported anxiety levels among those with persistent ED symptoms. However, given the modest effect size and confidence interval (OR = 0.92, 95% CI [0.85, 1.00]), this finding should be interpreted cautiously. The relationship between anxiety, rumination, and ED risk persistence appears more complex than simple additive effects and future studies are needed to understand these relationships.

These findings should be considered in the context of several limitations. Although it is highly sensitive and specific for suggesting a likely case of an ED [33], the SCOFF is not diagnostic and cannot distinguish between different types of EDs or the stage of illness. It has also demonstrated lower sensitivity for males compared to females, which may affect the accuracy of ED risk identification in male participants [86]. The SCOFF is only suitable for identifying probable cases of anorexia nervosa and bulimia nervosa and is unlikely to be effective for screening other types of EDs, such as binge eating disorder and avoidant/restrictive food intake disorder, which share similar prevalence rates, distress and impairments as anorexia nervosa in adolescents [87, 88]. Since the SCOFF cannot assess illness stage, it is possible that individuals with persistent risk were further along in their illness course on enrolment into this study, compared to those who initially showed risk but later improved. As the *Transitions* study was focussed on a transdiagnostic model of clinical staging and was not designed to specifically measure eating behaviours, formal diagnoses were not recorded. Therefore, our results are reflective of an increased risk of having any ED rather than the true prevalence of a specific classified disorder. Future studies should consider alternative assessment and screening measures that can identify a broader range of EDs across diagnostic categories and demonstrate better validity across sexes.

The study experienced considerable attrition, with only 494 of the initial 774 participants (63.82%) completing a follow-up ED risk assessment. Although there was no difference in attrition rates between those with high versus low ED risk at baseline, males and those with lower functioning were disproportionately lost to follow-up (see Supplementary Table S1). This differential attrition is consistent with broader patterns of disengagement from mental health care in these populations and resulted in a final sample with a higher proportion of females (68.83%). While this is consistent with the natural composition of help-seeking populations in youth mental health services [89, 90], it may limit generalisability to broader youth populations, particularly males and those with poorer functioning.

We acknowledge the limitation of not conducting comprehensive sex-specific analyses as a primary aim. While exploratory sex-stratified analyses were conducted post-hoc (see Supplementary Tables S3A and S3B), small male sample sizes (*n* = 30 for persistent versus remitting comparison) limited our ability to draw reliable conclusions about sex differences in predictor patterns. This is particularly important given that EDs present differently across sexes. Future research should be designed a priori with adequate power to examine sex-specific ED risk trajectories and their predictors using screening tools with demonstrated validity across sexes.

While this study offers valuable insights into the baseline clinical factors associated with ED risk trajectories that can be addressed through mental health care, it did not consider other well-established risk factors for eating disorders, such as genetics, personality traits, body image concerns and body dissatisfaction, and perfectionism [14]. These factors are known to play a significant role in the onset and persistence of eating disorders, and their exclusion from this analysis may have led to underestimation of true ED risk. Importantly, classification as “low risk” based on SCOFF scores does not indicate absence of all risk and individuals in the low risk group may still have elevated vulnerability based on other unmeasured risk factors. The exclusion of these factors limits the study’s ability to comprehensively explain ED risk trajectories. Additionally, unmeasured confounders such as treatment received during the follow-up period, body mass index, family history of eating disorders, and other environmental influences may have affected the observed relationships between baseline factors and trajectory outcomes. While the overall sample size was reasonable, smaller trajectory groups (particularly emerging risk, *n* = 51) may have limited power to detect associations with smaller effect sizes.

Finally, while this study examined longitudinal changes over time, having only two timepoints limited our ability to capture symptom patterns and fluctuations between baseline and follow-up and may have led to misclassification of trajectory patterns. Future research using digital technologies and ecological momentary assessments could enable more frequent monitoring and provide a more nuanced understanding of how symptoms change over time.

It is also important to highlight that the data for this study was collected in 2011-13, which may limit the direct applicability of some findings to contemporary youth populations. In the context of substantial sociocultural changes that have been linked to ED onset and maintenance, particularly the proliferation of social media platforms that give unfettered exposure to idealised body images and diet culture content [91], it is possible that the distribution and characteristics of risk trajectory patterns have evolved since our data collection period. Future longitudinal research using recently collected data is needed to examine whether trajectory patterns and their predictors are consistent with those reported here (and in previous studies).

This study identified distinct trajectories of ED risk among young people seeking mental health care. Just under one-third of participants experienced persistent or emerging ED risk, while the majority remained at low risk throughout the follow-up period. However, the considerable proportion showing persistent elevated risk highlights critical gaps in current treatment approaches within primary youth mental health care. Our findings demonstrated that rumination is associated with worse outcomes, suggesting its potential mechanistic role as a broader cognitive vulnerability driving ED risk. Longitudinal studies with longer follow-up periods are needed to better understand relapse patterns and identify factors that support sustained recovery.

## Supplementary Information

Below is the link to the electronic supplementary material.

Supplementary Material 1 (DOCX 30.8 KB)

## Data Availability

The data that support the findings of this study are not openly available due to reasons of sensitivity and are available from the corresponding author upon reasonable request.
