# Supplementary material for: Clinical characteristics linked to persistent and emerging eating disorder risk trajectories in young people seeking mental health care
Source: Eur Child Adolesc Psychiatry. 2026 Mar 17;35(6):1971–82. doi: 10.1007/s00787-026-02978-9 (PMC13337872; doi:10.1007/s00787-026-02978-9)
Supplement: Supplementary file 1 — Supplementary Material 1 (DOCX 30.8 KB) [file 787_2026_2978_MOESM1_ESM.docx]

In the sample who completed an ED risk assessment at baseline (n=774), differences in demographic and clinical variables between those included in the final sample (n=494) and those lost to follow-up (n=280) were assessed using Wilcoxon rank-sum test and Fisher’s exact test. Effect sizes and 95% confidence intervals were calculated to complement significance testing: rank biserial correlation coefficients (r) for continuous variables and odds ratios (OR) for categorical variables.

**Table S1. Demographic and clinical differences between the final sample and those lost to follow-up**

| **Characteristic** | **Included in final sample**  **(n=494)** | **Lost to follow-up**  **(n=280)** | **Effect size [95% CI]** | **p value** |
| --- | --- | --- | --- | --- |
| *Demographic variables* | |  |  |  |
| Age, years | 18.35 ± 3.21 | 18.20 ± 3.23 | r = 0.02 [-0.06, 0.10] | .640 |
| Sex, % female | 340 (68.83) | 171 (61.07) | OR = 0.71 [0.52, 0.98] | **.033** |
| NEET, % yes | 84 (17.00) | 53 (18.93) | OR = 1.14 [0.76, 1.69] | .495 |
|  |  |  |  |  |
| *Clinical variables* | |  |  |  |
| ED risk status, % high risk | 161 (32.59%) | 91 (32.50%) | OR = 1.00 [0.72, 1.38] | 1.00 |
| QIDS, score /27 | 10.14 ± 5.47 | 10.33 ± 5.10 | r = -0.02 [-0.11, 0.06] | .590 |
| GAD-7, score /21 | 9.73 ± 6.00 | 10.09 ± 5.95 | r = -0.04 [-0.12, 0.05] | .406 |
| SOFAS, score /100 | 66.37 ± 11.66 | 64.06 ± 11.38 | r = 0.11 [0.02, 0.19] | **.012** |
| Rumination, score /40^φ^ | 28.95 ± 6.83 | 28.71 ± 7.56 | r = 0.01 [-0.07, 0.09] | .811 |
| Deliberate self-harm, % yes | 212 (42.91) | 119 (42.50) | OR = 0.99 [0.73, 1.35] | .999 |

Note. NEET = Not in Education, Employment or Training; QIDS = Quick Inventory of Depressive Symptomatology; GAD = Generalised Anxiety Disorder; SOFAS = Social and Occupational Functioning Assessment Scale.

Data are presented as mean ± standard deviation for continuous variables and n (%) for categorical variables.

**Table S2. Changes in clinical variables from baseline to 12-month follow-up by ED risk trajectory group**

| **Risk trajectory group** | **Variable** | **Baseline** | **Follow-up** | **Mean change** |
| --- | --- | --- | --- | --- |
| *Persistent* | Depressive symptoms | 13.18 ± 5.34 | 11.02 ± 5.31 | -2.16 |
|  | Anxiety symptoms | 12.25 ± 6.02 | 10.09 ± 6.01 | -2.16 |
|  | Functioning | 62.72 ± 12.16 | 67.56 ± 13.63 | +4.84 |
|  |  |  |  |  |
| *Remitting* | Depressive symptoms | 11.00 ± 5.57 | 7.14 ± 5.17 | -3.86 |
|  | Anxiety symptoms | 11.68 ± 5.60 | 8.09 ± 5.38 | -3.59 |
|  | Functioning | 64.45 ± 11.00 | 70.41 ± 13.54 | +5.96 |
|  |  |  |  |  |
| *Emerging* | Depressive symptoms | 10.08 ± 5.07 | 10.59 ± 5.51 | +0.51 |
|  | Anxiety symptoms | 9.78 ± 6.37 | 8.78 ± 5.56 | -1.00 |
|  | Functioning | 66.25 ± 11.48 | 68.06 ± 12.11 | +1.80 |
|  |  |  |  |  |
| *Low* | Depressive symptoms | 8.95 ± 5.15 | 6.56 ± 4.90 | -2.39 |
|  | Anxiety symptoms | 8.41 ± 5.65 | 5.87 ± 5.41 | -2.55 |
|  | Functioning | 68.05 ± 11.38 | 72.10 ± 12.85 | +4.05 |

Note. Positive change scores indicate symptom worsening for depressive and anxiety symptoms, while positive change scores for functioning indicate improvement.

Data are presented as mean ± standard deviation.

Results of exploratory sex-stratified logistic regression analyses.

**Table S3A. Demographic and clinical characteristics associated with risk trajectories in females**

| **Factor** | **Persistent vs remitting risk^#^**  **(n=131)** | | **Emerging vs low risk^^^**  **(n=209)** | |
| --- | --- | --- | --- | --- |
|  | *OR [95% CI]* | *p value* | *OR [95% CI]* | *p value* |
| Age | 1.12 [0.99, 1.20] | .086 | 0.86 [0.75, 0.97] | **.019** |
| Depressive symptoms | 1.10 [0.99, 1.23] | .072 | 0.99 [0.90, 1.09] | .785 |
| Anxiety symptoms | 0.88 [0.80, 0.97] | **.009** | 0.97 [0.90, 1.05] | .509 |
| Functioning | 1.00 [0.96, 1.04] | .976 | 0.98 [0.95, 1.02] | .387 |
| Rumination | 1.08 [1.00, 1.17] | .068 | 1.11 [1.03, 1.20] | **.012** |
| Deliberate self-harm (Ref: No) | 1.94 [0.88, 4.33] | .102 | 1.00 [0.47, 2.10] | .996 |

**Table S3B. Demographic and clinical characteristics associated with risk trajectories in males**

| **Factor** | **Persistent vs remitting risk^#^**  **(n=30)** | | **Emerging vs low risk^^^**  **(n=124)** | |
| --- | --- | --- | --- | --- |
|  | *OR [95% CI]* | *p value* | *OR [95% CI]* | *p value* |
| Age | 0.55 [0.26, 0.88] | **.047** | 0.99 [0.83, 1.18] | .917 |
| Depressive symptoms | 0.85 [0.50, 1.27] | .462 | 0.99 [0.83, 1.17] | .882 |
| Anxiety symptoms | 1.11 [0.84, 1.50] | .452 | 1.01 [0.86, 1.18] | .890 |
| Functioning | 0.86 [0.67, 1.01] | .131 | 1.00 [0.94, 1.06] | .948 |
| Rumination | 2.08 [1.26, 5.23] | **.031** | 1.03 [0.91, 1.16] | .643 |
| Deliberate self-harm (Ref: No) | 0.08 [0.01, 1.82] | .164 | 0.77 [0.16, 2.88] | .717 |

Note. ED = eating disorder; OR = Odds Ratio; CI = Confidence Interval. Depressive symptoms measured using Quick Inventory of Depressive Symptomatology scale (QIDS); Anxiety symptoms measured using Generalised Anxiety Disorder scale (GAD-7); Rumination measured using brief rumination questionnaire; Functioning measured using Social and Occupational Functioning Assessment Scale (SOFAS).

^#^Persistent vs remitting risk compares those with persistent risk to those whose high risk remitted. Reference group: Remitting risk.

^^^Emerging vs no risk compares those who developed high ED risk to those who maintained low risk. Reference group: Low risk.

Significant factors (*p*<.05) are shown in bold.
